# Association of Pre- and Posttreatment Neutrophil–Lymphocyte Ratio With Recurrence and Mortality in Locally Advanced Non-Small Cell Lung Cancer

**DOI:** 10.3389/fonc.2020.598873

**Published:** 2020-11-05

**Authors:** Nikhil T. Sebastian, Rohit Raj, Rahul Prasad, Christian Barney, Jeremy Brownstein, John Grecula, Karl Haglund, Meng Xu-Welliver, Terence M. Williams, Jose G. Bazan

**Affiliations:** ^1^Department of Radiation Oncology, The Ohio State University Comprehensive Cancer Center—Arthur G. James Cancer Hospital and Richard J. Solove Research Institute, Columbus, OH, United States; ^2^Intermountain Radiation Oncology, Provo, UT, United States

**Keywords:** neutrophil–lymphocyte ratio, chemoradiation, locally advanced, non-small cell lung cancer, vertebral body

## Abstract

**Objectives:**

Neutrophil–lymphocyte ratio (NLR) has been associated with mortality in non-small cell lung cancer (NSCLC), but its association with recurrence in locally advanced NSCLC (LA-NSCLC), specifically, is less established. We hypothesized pre- and posttreatment NLR would be associated with recurrence and mortality.

**Methods:**

We studied the association of pretreatment NLR (pre-NLR) and posttreatment NLR at 1 (post-NLR_1_) and 3 months (post-NLR_3_) with outcomes in patients with LA-NSCLC treated with chemoradiation. Pre-NLR was dichotomized by 5, an *a priori* cutoff previously shown to be prognostic in LA-NSCLC. Post-NLR_1_ and post-NLR_3_ were dichotomized by their medians.

**Results:**

We identified 135 patients treated with chemoradiation for LA-NSCLC between 2007 and 2016. Median follow-up for living patients was 61.1 months. On multivariable analysis, pre-NLR ≥ 5 was associated with worse overall survival (HR = 1.82; 95% CI 1.15 – 2.88; p = 0.011), but not with any recurrence, locoregional recurrence, or distant recurrence. Post-NLR_1_ ≥ 6.3 was not associated with recurrence or survival. Post-NLR_3_ ≥ 6.6 was associated with worse overall survival (HR = 3.27; 95% CI 2.01– 5.31; p < 0.001), any recurrence (HR = 2.50; 95% CI 1.53 – 4.08; p < 0.001), locoregional recurrence (HR = 2.50; 95% CI 1.40 – 4.46; p = 0.002), and distant recurrence (HR = 2.53; 95% CI 1.49 – 4.30; p < 0.001).

**Conclusion:**

Pretreatment NLR is associated with worse overall survival and posttreatment NLR is associated with worse survival and recurrence. These findings should be validated independently and prospectively studied.

## Introduction

Chemoradiotherapy remains the standard of care for unresectable locally advanced non-small cell lung cancer (NSCLC), and despite substantial improvements with the integration of immunotherapy, the majority of patients with locally advanced NSCLC will have disease progression or die from disease within two years (1, 2). Identification of novel prognostic biomarkers may allow for improved stratification of patients for therapy escalation or tailoring follow-up.

Neutrophil–lymphocyte ratio (NLR) has emerged as an impactful prognostic biomarker in a variety of malignancies, in part attributed to its quantification of inflammation, which appears to play a major role in oncogenesis and cancer progression (3). Multiple studies suggest that pretreatment NLR is prognostic in NSCLC, potentially predicting for both overall and progression-free survival, and a meta-analysis identified a cutoff value of 5 as consistently prognostic (4). Despite this, there are limited data regarding NLR in the setting of locally advanced NSCLC treated with chemoradiation. A recent study identified an association of overall survival with NLR in locally advanced NSCLC validated a threshold NLR of 5 as prognostic (5). No studies have evaluated the prognostic utility of posttreatment NLR in the setting of locally advanced NSCLC; whereas pretreatment NLR is a manifestation of baseline immune function, posttreatment NLR is a theoretically modifiable factor that could be influenced by a number of factors, such as radiation prescription or dose to at-risk organs. Thus, we sought to independently validate the prognostic utility of pretreatment NLR (pre-NLR) in the setting of chemoradiation for locally advanced NSCLC. Additionally, we sought to determine the prognostic utility of posttreatment NLR obtained at 1 month (post-NLR_1_) and 3 months (post-NLR_3_) after completion of chemoradiation.

## Materials and Methods

### Patient Selection

This retrospective study was approved by the Institutional Review Board at The Ohio State University James Cancer Hospital. We included all patients with locally advanced (American Joint Committee on Cancer 7th edition stage III), biopsy-proven NSCLC treated with definitive concurrent chemotherapy and radiation between 2007 and 2016 at our institution. We included only patients with available pretreatment NLR obtained from a complete blood count (CBC) with differential.

### Staging and Treatment

All patients were staged with positron emission tomography (PET) and brain magnetic resonance imaging (MRI). Bronchoscopy and pathologic mediastinal node evaluation was also performed to confirm PET-avid mediastinal lymph nodes except in patients deemed too high risk for this procedure. For radiation treatment planning, free-breathing and four-dimensional (4D) computed tomography (CT) simulation scans were obtained with or without contrast. The gross tumor volume (GTV) was contoured on the free-breathing scan and internal target volumes (ITVs) were generated using the 4D scan. Clinical target volumes (CTVs) were created by expanding the ITV by 5–10 mm and cropping from barriers to spread, and planning target volumes (PTVs) were created with a subsequent 5–10 mm expansion of the CTV. The range of total delivered radiation dose was 50–70 Gy (interquartile range 60–63 Gy).

Patients were treated with either concurrent cisplatin 50 mg/m^2^ on days 1, 8, 29, 36 and etoposide 50 mg/m^2^ on days 1–5 and 29–33, or weekly carboplatin (area under the plasma concentration time curve [AUC] = 2) and paclitaxel (45 mg/m^2^) per discretion of the medical oncologist. After chemoradiation, patients were followed with interval history and physical and CT chest every 3–6 months for 3 years, then every 6 months for 2 years, and annually thereafter.

### Statistical Analysis

Pre-NLR was obtained from the most recent CBC obtained within 1-2 weeks prior to starting chemoradiation and calculated as the quotient of absolute neutrophil count divided by absolute lymphocyte count. Additionally, posttreatment NLR at 1 month and 3 months after treatment were calculated. Patient characteristics were compared using Pearson chi-square for categorical variables and unpaired t-test or Wilcoxon rank sum (Mann-Whitney U) tests for continuous variables. Differences between pre-NLR, post-NLR_1_, and post-NLR_3_ were compared using Wilcoxon signed rank test, with a Bonferroni corrected α of 0.05/3 = 0.017 for multiple comparisons. Pre- and posttreatment NLR were analyzed both as continuous and dichotomized categorical variables. An *a priori* cutoff value of pre-NLR ≥ 5 was used to analyze NLR as a dichotomized variable on the basis of a prior study identifying this cutoff as prognostic in locally advanced NSCLC (5). For post-NLR_1_ and post-NLR_3_, the median posttreatment NLR value was used as the cutoff for dichotomization.

## Results

### Pretreatment NLR as a Prognostic Biomarker

We identified 135 patients treated with chemoradiation for locally advanced NSCLC treated between 2007 and 2016 with available hematological data and follow-up. **Supplementary Figure 1** shows a flowchart of how patients were selected for study. The median dose of radiation was 60 Gy (IQR 60–63 Gy). The median pre-NLR for the entire cohort was 4.2 (IQR 2.5–6.8) and the median time from pre-NLR to treatment was 0 days (IQR 0–3 days). **Table 1** shows the patient and disease characteristics of the entire cohort, stratified by pre-NLR < or ≥ 5. Pre-NLR ≥ 5 was associated with older age (p < 0.001), higher proportion of males (p = 0.01), and worse ECOG performance status (p = 0.01).

**Table 1:** Patient characteristics of patients with pretreatment NLR < or ≥5*.

Variable	All patients(N = 135)	NLR < 5(N = 91)	NLR ≥ 5(N = 44)	P value^†^
**Pretreatment NLR**				
Median (IQR)	4.2 (2.5–6.9)	2.8 (2.2–4.2)	8.8 (6.9–12.1)	<.001
**Age**				
Median (IQR)	64 (56–71)	61 (55–69)	69 (61–73)	<.001
**Sex**				
Male	74 (54.8%)	43 (47.3%)	31 (70.5%)	0.01
Female	61 (45.2%)	48 (52.7%)	13 (29.5%)	
**ECOG performance status**				
0	43 (31.9%)	36 (39.6%)	7 (15.9%)	
1	81 (60.0%)	47 (51.6%)	34 (77.3%)	0.01
2	11 (8.1%)	8 (8.8%)	3 (6.8%)	
**Histology**				
Squamous cell carcinoma	66 (48.9%)	40 (44.0%)	26 (59.1%)	
Adenocarcinoma	60 (44.4%)	45 (49.5%)	15 (34.1%)	0.23
NSCLC NOS	9 (6.7%)	6 (6.6%)	3 (6.8%)	
**Clinical stage^‡^**				
IIIA	92 (68.1%)	66 (72.5%)	26 (59.1%)	0.12
IIIB	43 (31.9%)	26 (27.5%)	18 (40.9%)	
**Radiation dose**				
Median (IQR)	60 (60–63)	60 (60–63)	60 (60–63)	0.70
**Concurrent chemotherapy**				
Cisplatin/etop oside	59 (43.7%)	43 (47.3%)	16 (36.4%)	0.23
Carboplatin/paclitaxel	76 (56.3%)	48 (52.7%)	28 (63.6%)	
**Induction chemotherapy**				
No	123 (91.1%)	84 (92.3%)	39 (88.6%)	0.48
Yes	12 (8.9%)	7 (7.7%)	5 (11.4%)	

*NLR, neutrophil–lymphocyte ratio; ECOG, Eastern Cooperative Oncology Group; NSCLC, non-small cell lung cancer, not otherwise specified; NSCLC NOS, non-small cell lung cancer not otherwise specified.

^†^Pretreatment NLR and radiation dose were tested by Mann–Whitney U test, Age was tested by independent t-test, and the remaining discrete variables were tested by Pearson chi square test.

^††^American Joint Committee on Cancer (AJCC) 7th edition.

**Figure 1:**
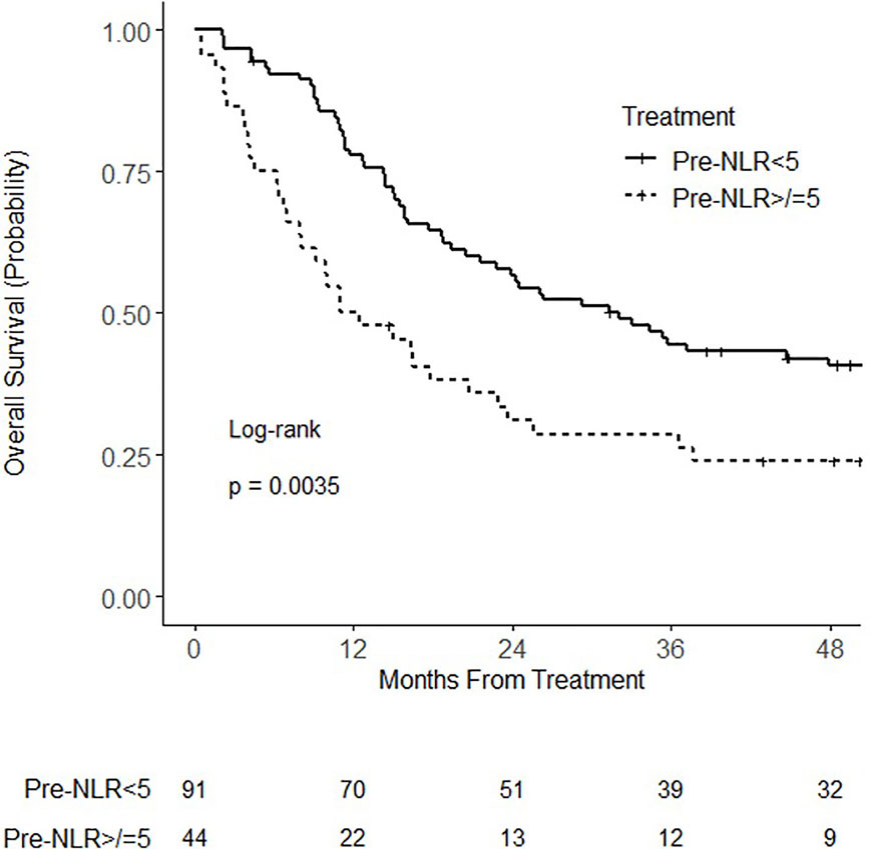
Kaplan–Meier curves for overall survival for patients with pretreatment neutrophil–lymphocyte ratio (NLR) (pre-NLR) <5 or ≥5.

At the time of analysis, 94 patients had died. Median follow-up for all patients was 22.8 months (IQR 10.3–53.5 months) and for living patients was 61.1 months (IQR 49.5–71.5 months). When analyzed as a continuous variable, pre-NLR was not associated with overall survival on univariate (HR = 1.03; 95% CI 0.99–1.06; p = 0.13) or multivariable analysis (HR = 1.02; 95% CI 0.99–1.06; p = 0.21). When analyzed as a categorical variable, pre-NLR ≥ 5 was associated with worse overall survival on univariate (HR = 1.87; 95% CI 1.22 – 2.86; p = 0.004) (**Figure 1**) and multivariable (HR = 1.82; 95% CI 1.15–2.88; p = 0.011) analysis (**Table 2**). There was no statistically significant association of overall survival with age, ECOG performance status, sex, clinical stage, histology, or type of concurrent chemotherapy.

**Table 2:** Univariate and multivariable analyses for overall survival*.

Variable	Univariate		Multivariable	
	HR (95% CI)	P	HR (95% CI)	P
**Pretreatment NLR**				
<5	1.0	–	1.0	–
≥5	1.87 (1.22–2.86)	0.004	1.82 (1.15–2.88)	0.011
**Age**	1.01 (0.99–1.03)	0.31	1.01 (0.98–1.03)	0.60
**Sex**				
Male	1.0	–	1.0	–
Female	0.74 (0.49–1.11)	0.15	0.83 (0.53–1.31)	0.42
**Clinical Stage**^†^				
IIIA	1.0	–	1.0	–
IIIB	1.21 (0.79–1.86)	0.37	1.28 (0.82–2.00)	0.28
**Histology**				
SCC	1.0	–	1.0	–
Adenocarcinoma	1.05 (0.69–1.60)	0.81	1.18 (0.76–1.83)	0.47
NSCLC, NOS	0.91 (0.38–2.15)	0.82	0.99 (0.41–2.40)	0.98
**ECOG**				
0–1	1.0	–	1.0	–
2–3	1.46 (0.70–3.03)	0.31	1.61 (0.73–3.52)	0.24
**Chemotherapy**				
Cisplatin/etoposide	1.0	–	1.0	–
Carboplatin/paclitaxel	1.31 (0.87–1.98)	0.32	1.26 (0.79–2.01)	0.34

*HR, hazard ratio; CI, confidence interval; NLR, neutrophil–lymphocyte ratio; SCC, squamous cell carcinoma; NSCLC, NOS, non-small cell lung cancer, not otherwise specified; ECOG, Eastern Cooperative Oncology Group.

^†^American Joint Committee on Cancer (AJCC) 7th edition.

**Figure 2:**
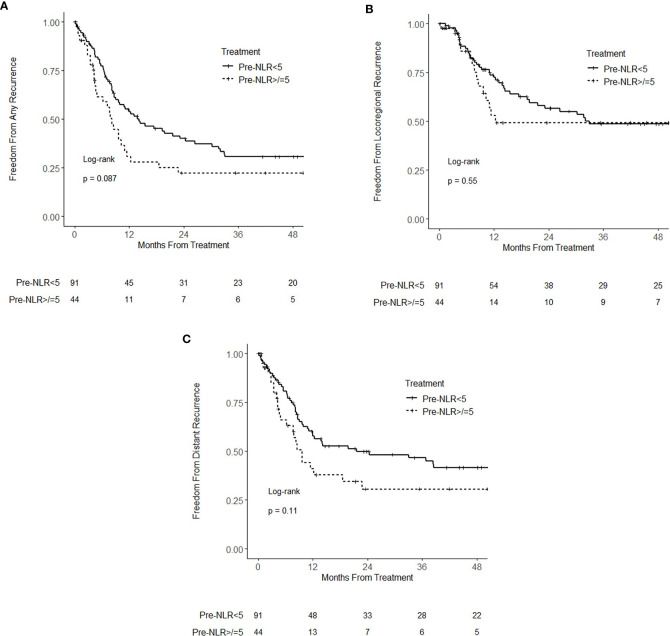
Kaplan–Meier curves for **(A)** freedom from recurrence **(B)** freedom from locoregional recurrence, and **(C)** freedom from distant recurrence, stratified by pretreatment neutrophil–lymphocyte ratio (NLR) (pre-NLR) <5 and ≥5.

At the time of analysis, there were 92 recurrence events (16 locoregional only, 32 distant only, 44 locoregional and distant). On univariate analysis, pre-NLR, analyzed as a continuous variable, was significantly associated with any recurrence (HR = 1.04; 95% CI 1.01–1.08; p = 0.021) and distant recurrence (HR = 1.04; 95% CI 1.01–1.08; p = 0.023). There was no statistically significant association of pre-NLR with locoregional recurrence (HR 0.99; 95% CI 0.93–1.04; p = 0.65). On multivariable analysis, pretreatment NLR as a continuous variable was not associated with any recurrence (p = 0.10), locoregional recurrence (p = 0.57), or distant recurrence (p = 0.14). When analyzed as a categorical variable, pre-NLR ≥ 5 was not associated with any recurrence (HR = 1.47; 95% CI 0.94–2.29; p = 0.089), locoregional recurrence (HR = 1.19; 95% CI 0.67–2.12; p = 0.55) or distant recurrence (HR = 1.48; 95% CI 0.91–2.42; p = 0.11) (**Figure 2**). On multivariable analysis, there was no statistically significant association of pre-NLR ≥ 5 with any recurrence (HR = 1.39; 95% CI 0.87–2.24; p = 0.17), locoregional recurrence (HR = 1.09; 95% CI 0.59–2.03; p = 0.78), or distant recurrence (HR = 1.54; 95% CI 0.92–2.59; p = 0.10). None of the other analyzed variables had a statistically significant association with locoregional recurrence (**Supplementary Table 1**). Clinical stage IIIB and non-squamous cell carcinoma histology were associated with distant recurrence (**Supplementary Table 2**).

### Posttreatment NLR as a Prognostic Biomarker

Of the 135 patients, 125 and 117 were evaluable for post-NLR at 1-month and 3-months, respectively. The median NLR at 1 month and 3 months posttreatment was 6.3 (IQR 3.8–9.4) and 6.6 (IQR 4.2–10.5), respectively. When analyzing posttreatment NLR, there was a statistically significant increase in NLR at 1 month posttreatment (p < 0.001) and 3 months posttreatment (p < 0.001) compared to pretreatment NLR. There was also a statistically significant increase in NLR at 3 months posttreatment compared to 1 month (p = 0.014) (**Supplementary Figure 2**).

**Supplementary Tables 3** and **4** show the univariate and multivariable hazard ratios for post-NLR_1_ analyzed as a continuous and dichotomized (< or ≥ 6.3) variable, respectively. **Supplementary Figure 3** shows the Kaplan Meier curves for recurrence and survival for post-NLR_1_ < 6.3 versus ≥ 6.3. On multivariable analysis, post-NLR_1_ (analyzed as a continuous variable) was associated with worse overall survival (p < 0.001) and any recurrence (p = 0.047). On multivariable analysis, post-NLR_1_ ≥ 6.3 was not significantly associated with any endpoint.

**Figure 3:**
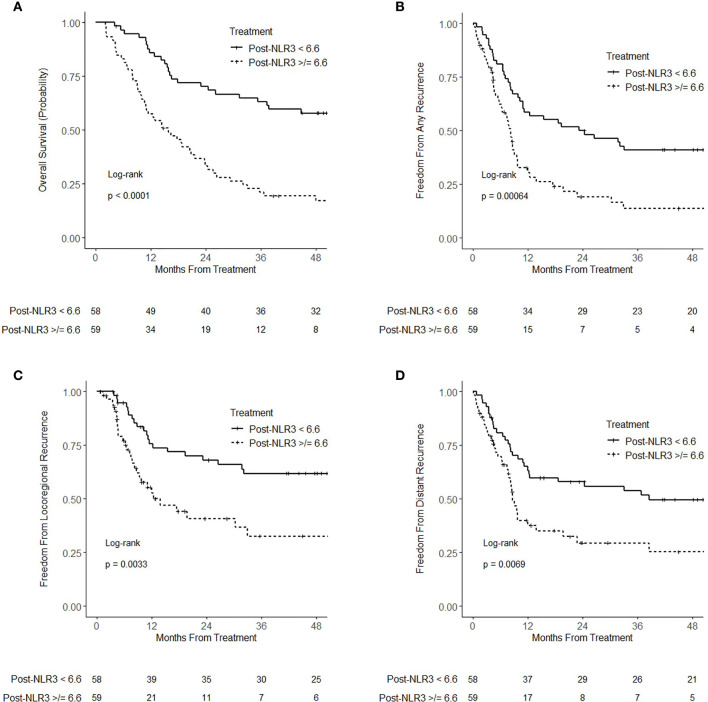
Kaplan–Meier curves for **(A)** overall survival **(B)** freedom from recurrence **(C)** freedom from locoregional recurrence, and **(D)** freedom from distant recurrence, stratified by 3-month-posttreatment neutrophil–lymphocyte ratio (NLR) (post-NLR_3_) <6.6 and ≥6.6.

Post-NLR_3_ analyzed as a continuous variable was associated with worse overall survival (HR = 1.10; 95% CI 1.07–1.14; p < 0.001), any recurrence (HR = 1.10; 95% CI 1.06–1.14; p < 0.001), locoregional recurrence (HR = 1.05; 95% CI 1.01–1.09; p = 0.011), and distant recurrence (HR = 1.04; 95% CI 1.01–1.07; p = 0.021). On multivariable analysis, post-NLR_3_ remained associated with worse overall survival (HR = 1.06; 95% CI 1.04–1.09; p < 0.001), any recurrence (HR = 1.10; 95% CI 1.06–1.14; p < 0.001), locoregional recurrence (HR = 1.07; 95% CI 1.04–1.11; p < 0.001), and distant recurrence (HR = 1.09; 95% CI 1.05–1.13; p < 0.001) (**Supplementary Table 5**). On multivariable analysis including pre-NLR, post-NLR_3_ was significantly associated with overall survival (HR = 1.11; 95% CI 1.07–1.14; p < 0.001) and any recurrence (HR = 1.10; 95% CI 1.06–1.14; p < 0.001), while pre-NLR was not statistically significant (p = 0.50 and p = 0.09, respectively). Analyzed as a dichotomized categorical variable using median post-NLR_3_, post-NLR_3_ ≥ 6.6 was associated with worse overall survival (HR = 3.04; 95% CI 1.91–4.84; p < 0.001), any recurrence (HR = 2.16; 95% CI 1.37–3.38; p < 0.001), locoregional recurrence (HR = 2.21; 95% CI 1.29–3.81; p = 0.004), and distant recurrence (HR = 1.93; 95% CI 1.19–3.13; p = 0.008) (**Figures 3A–D**). On multivariable analysis, post-NLR_3_ ≥ 6.6 remained associated with worse overall survival (HR = 3.27; 95% CI 2.01–5.31; p < 0.001), any recurrence (HR = 2.50; 95% CI 1.53–4.08; p < 0.001), locoregional recurrence (HR = 2.50; 95% CI 1.40–4.46; p = 0.002), and distant recurrence (HR = 2.53; 95% CI 1.49–4.30; p < 0.001) (**Supplementary Table 6**).

## Discussion

In our study, we validated the prognostic utility of pretreatment NLR (and specifically a threshold of NLR ≥ 5) for overall survival in patients receiving chemoradiation for locally advanced NSCLC. Additionally, for the first time, we identified that posttreatment NLR 3 months after chemoradiation is strongly associated with higher rate of mortality, locoregional recurrence, and distant recurrence.

In a pooled meta-analysis of studies evaluating NLR in NSCLC, pretreatment NLR was found to correlate with overall survival and progression-free survival in patients with NSCLC, and a subgroup analysis identified an NLR cutoff value of 5 as most optimal for prognostication (4). This study contained NSCLC of all stages and did not stratify for association on the basis of stage. A subsequent study in a cohort of patients treated with chemoradiation for NSCLC found that pretreatment NLR is prognostic for overall survival, particularly when dichotomized by a value of 5 (5). This study, however, did not assess for correlation with disease recurrence. In contrast, our study specifically evaluated a cohort of locally advanced NSCLC patients treated with chemoradiation and evaluated the association of pretreatment NLR with overall survival, locoregional recurrence, and distant recurrence. The prognostic value of NLR has been attributed to its ability to stratify comorbidity and chronic inflammation, in part based on its association with mortality in benign conditions such as cardiovascular and renal disease (6–8). Given that NLR may be associated with disease recurrence, it has been hypothesized that there may be interplay between NLR and tumor control and that NLR may be a surrogate for systemic inflammatory responses that promote tumor progression or resistance to therapy (9, 10). In the context of pretreatment NLR, our study does not support this link, although the lack of a statistically significant association of pretreatment NLR with recurrence may be a function of inadequate statistical power.

Post-radiotherapy NLR has been shown to be prognostic in a variety of solid tumors (11–14), but its capacity for predicting recurrence and mortality has not previously been evaluated in the setting of chemoradiation for locally advanced NSCLC. We found posttreatment NLR at 3 months is highly associated with mortality and recurrence. Thus, an elevated 3-month posttreatment NLR after treatment could be used to potentially dictate closer follow up or addition of consolidative therapies. Additionally, we found that NLR increases after radiation, a finding that has been identified in other studies (15–17) and possibly reflects a sustained inflammatory response to radiotherapy and/or depletion of lymphocytes, which are highly radiosensitive. It is possible that the lack of significant association of posttreatment NLR at 1 month with outcomes is due to a more acute inflammatory or lymphodepleted state not indicative of the chronic immunologic changes that are more likely to influence cancer recurrence. Future studies should evaluate whether post-NLR is a potentially modifiable factor. In particular, vertebral body dose has previously been linked to hematologic toxicity in lung cancer patients receiving chemoradiation, (18) and it would be interesting to correlate dose-volume analysis of thoracic vertebral bodies (for which dose is not typically assessed) with posttreatment NLR.

There are limitations of this study that should be noted in addition to its retrospective design and potential for selection bias. First, none of the patients in this study were treated with consolidative immunotherapy, the current standard of care for locally advanced NSCLC without progression on chemoradiotherapy (2). However, pretreatment NLR is a known marker of outcomes in metastatic NSCLC treated with immunotherapy, (19) so it is plausible that findings similar to those found in our study would be expected in an identical cohort treated with consolidative durvalumab. Additionally, although we account for performance status, we did not account for comorbidities or medication use (e.g. steroids), which could theoretically affect NLR. The inclusion of patients who received induction chemotherapy may also confound NLR pretreatment values. There are also known treatment-related variables that we did not account for that have been shown to be associated with lymphopenia, such as lung (and possibly heart) V_5_ (20), which may influence posttreatment NLR.

In summary, we validated that pretreatment NLR ≥ 5 is associated with overall mortality in locally advanced NSCLC patients treated with chemoradiation. Additionally, we found that elevated posttreatment NLR at 3 months is highly associated with overall mortality and recurrence. Thus, NLR could potentially be used for prognostication as well as tailoring escalation of treatment or follow up. Further studies are needed to validate our findings.

## Data Availability Statement

The data analyzed in this study is subject to the following licenses/restrictions: institutional data restricted by IRB. Requests to access these datasets should be directed to Jose.Bazan2@osumc.edu.

## Ethics Statement

This retrospective study was approved by the Institutional Review Board at The Ohio State University James Cancer Hospital.

## Author Contributions

NS was involved in the conception and design, data collection, analysis, and writing. JB and TW were involved in the conception and design and writing. RR was involved in the data collection and writing. All remaining authors were involved in writing. All authors contributed to the article and approved the submitted version.

## Conflict of Interest

The authors declare that the research was conducted in the absence of any commercial or financial relationships that could be construed as a potential conflict of interest.

## References

[1] Bradley JD Paulus R Komaki R Masters G Blumenschein G Schild S Standard-dose versus high-dose conformal radiotherapy with concurrent and consolidation carboplatin plus paclitaxel with or without cetuximab for patients with stage iiia or iiib non-small-cell lung cancer (rtog 0617): A randomised, two-by-two factorial phase 3 study. Lancet Oncol (2015) 16:187–99. 10.1016/S1470-2045(14)71207-0 PMC4419359 25601342

[2] Antonia SJ Villegas A Daniel D Vicente D Murakami S Hui R Durvalumab after chemoradiotherapy in stage iii non-small-cell lung cancer. N Engl J Med (2017) 377:1919–29. 10.1056/NEJMoa1709937 28885881

[3] Templeton AJ McNamara MG Seruga B Vera-Badillo FE Aneja P Ocana A Prognostic role of neutrophil-to-lymphocyte ratio in solid tumors: A systematic review and meta-analysis. J Natl Cancer Inst (2014) 106:dju124. 10.1093/jnci/dju124 24875653

[4] Gu XB Tian T Tian XJ Zhang XJ Prognostic significance of neutrophil-to-lymphocyte ratio in non-small cell lung cancer: A meta-analysis. Sci Rep (2015) 5:12493. 10.1038/srep12493 26205001 PMC4513342

[5] Scilla KA Bentzen SM Lam VK Mohindra P Nichols EM Vyfhuis MA Neutrophil-lymphocyte ratio is a prognostic marker in patients with locally advanced (stage iiia and iiib) non-small cell lung cancer treated with combined modality therapy. Oncologist (2017) 22:737–42. 10.1634/theoncologist.2016-0443 PMC5469587 28533476

[6] Pourafkari L Choi C Garajehdaghi R Tajlil A Dosluoglu HH Nader ND Neutrophil-lymphocyte ratio is a marker of survival and cardiac complications rather than patency following revascularization of lower extremities. Vasc Med (2018) 23:437–44. 10.1177/1358863X18774623 29848209

[7] Duffy BK Gurm HS Rajagopal V Gupta R Ellis SG Bhatt DL Usefulness of an elevated neutrophil to lymphocyte ratio in predicting long-term mortality after percutaneous coronary intervention. Am J Cardiol (2006) 97:993–6. 10.1016/j.amjcard.2005.10.034 16563903

[8] Ouellet G Malhotra R Penne EL Usvya L Levin NW Kotanko P Neutrophil-lymphocyte ratio as a novel predictor of survival in chronic hemodialysis patients. Clin Nephrol (2016) 85:191–8. 10.5414/CN108745 26951970

[9] Hanahan D Weinberg RA Hallmarks of cancer: The next generation. Cell (2011) 144:646–74. 10.1016/j.cell.2011.02.013 21376230

[10] Mantovani A Allavena P Sica A Balkwill F Cancer-related inflammation. Nature (2008) 454:436–44. 10.1038/nature07205 18650914

[11] Chowdhary M Switchenko JM Press RH Jhaveri J Buchwald ZS Blumenfeld PA Post-treatment neutrophil-to-lymphocyte ratio predicts for overall survival in brain metastases treated with stereotactic radiosurgery. J Neurooncol (2018) 139:689–97. 10.1007/s11060-018-2914-5 PMC6126986 29846893

[12] Lin AJ Gang M Rao YJ Campian J Daly M Gay H Association of posttreatment lymphopenia and elevated neutrophil-to-lymphocyte ratio with poor clinical outcomes in patients with human papillomavirus-negative oropharyngeal cancers. JAMA Otolaryngol Head Neck Surg (2019) 145:413–21. 10.1001/jamaoto.2019.0034 PMC6537794 30920592

[13] Choi N Kim JH Chie EK Gim J Kang HC A meta-analysis of the impact of neutrophil-to-lymphocyte ratio on treatment outcomes after radiotherapy for solid tumors. Med (Baltimore) (2019) 98:e15369. 10.1097/MD.0000000000015369 PMC6504242 31045780

[14] Ohno Y Nakashima J Ohori M Gondo T Hatano T Tachibana M Followup of neutrophil-to-lymphocyte ratio and recurrence of clear cell renal cell carcinoma. J Urol (2012) 187:411–7. 10.1016/j.juro.2011.10.026 22177153

[15] D’Emic N Engelman A Molitoris J Hanlon A Sharma NK Moeslein FM Prognostic significance of neutrophil-lymphocyte ratio and platelet-lymphocyte ratio in patients treated with selective internal radiation therapy. J Gastrointest Oncol (2016) 7:269–77. 10.3978/j.issn.2078-6891.2015.108 PMC4783753 27034796

[16] Sung S Son SH Park EY Kay CS Prognosis of locally advanced rectal cancer can be predicted more accurately using pre- and post-chemoradiotherapy neutrophil-lymphocyte ratios in patients who received preoperative chemoradiotherapy. PLoS One (2017) 12:e0173955. 10.1371/journal.pone.0173955 28291841 PMC5349688

[17] Sebastian N Wu T Bazan J Driscoll E Willers H Yegya-Raman N Pre-treatment neutrophil-lymphocyte ratio is associated with overall mortality in localized non-small cell lung cancer treated with stereotactic body radiotherapy. Radiother Oncol (2019) 134:151–7. 10.1016/j.radonc.2019.01.032 PMC10905623 31005209

[18] Barney CL Scoville N Allan E Ayan A DiCostanzo D Haglund KE Radiation dose to the thoracic vertebral bodies is associated with acute hematologic toxicities in patients receiving concurrent chemoradiation for lung cancer: Results of a single-center retrospective analysis. Int J Radiat Oncol Biol Phys (2018) 100:748–55. 10.1016/j.ijrobp.2017.11.025 PMC7193687 29413286

[19] Bagley SJ Kothari S Aggarwal C Bauml JM Alley EW Evans TL Pretreatment neutrophil-to-lymphocyte ratio as a marker of outcomes in nivolumab-treated patients with advanced non-small-cell lung cancer. Lung Cancer (2017) 106:1–7. 10.1016/j.lungcan.2017.01.013 28285682

[20] Chen D Patel RR Verma V Ramapriyan R Barsoumian HB Cortez MA Interaction between lymphopenia, radiotherapy technique, dosimetry, and survival outcomes in lung cancer patients receiving combined immunother. Radiother Oncol (2020) 150:114–20. 10.1016/j.radonc.2020.05.051 32525003

