# Supplementary material for: Association of Pre- and Posttreatment Neutrophil–Lymphocyte Ratio With Recurrence and Mortality in Locally Advanced Non-Small Cell Lung Cancer
Source: Front Oncol. 2020 Nov 5;10:598873. doi: 10.3389/fonc.2020.598873 (PMC7676908; doi:10.3389/fonc.2020.598873)

**Supplementary Table 1.** Pre-treatment univariate and multivariable analyses for locoregional recurrence.*

|  | **Univariate** | | **Multivariable** | |
| --- | --- | --- | --- | --- |
| **Variable** | **HR (95% CI)** | **P** | **HR (95% CI)** | **P** |
| **Pre-treatment NLR**  < 5  ≥ 5 | 1.0  1.19 (0.67 – 2.12) | --  0.55 | 1.0  1.09 (0.59 – 2.03) | --  0.78 |
| **Age** | 1.00 (0.97 – 1.02) | 0.75 | 1.00 (0.97 – 1.03) | 0.99 |
| **Sex**  Male  Female | 1.0  0.75 (0.45 – 1.26) | --  0.28 | 1.0  0.76 (0.43 – 1.34) | --  0.34 |
| **Clinical Stage**^†^  IIIA  IIIB | 1.0  1.23 (0.72 – 2.09) | --  0.45 | 1.0  1.27 (0.72 – 2.23) | --  0.41 |
| **Histology**  SCC  Adenocarcinoma  NSCLC, NOS | 1.0  0.72 (0.43 – 1.22)  0.73 (0.17 – 3.16) | --  0.22  0.12 | 1.0  0.82 (0.47 – 1.41)  0.30 (0.07 – 1.32) | --  0.47  0.11 |
| **ECOG**  0-1  2-3 | 1.0  2.22 (0.94 – 5.22) | --  0.068 | 1.0  1.99 (0.75 – 5.24) | --  0.17 |
| **Chemotherapy**  Cisplatin/etoposide  Carboplatin/paclitaxel | 1.0  0.97 (0.58 – 1.62) | --  0.91 | 1.0  0.92 (0.50 – 1.67) | --  0.78 |

*Abbreviations: HR- hazard ratio. CI- confidence interval. NLR- neutrophil-lymphocyte ratio. SCC- squamous cell carcinoma. NSCLC, NOS- non-small cell lung cancer, not otherwise specified. ECOG- Eastern Cooperative Oncology Group.

† American Joint Committee on Cancer (AJCC) 7^th^ edition.

**Supplementary Table 2.** Pre-treatment NLR univariate and multivariable analyses for distant recurrence.*

|  | **Univariate** | | **Multivariable** | |
| --- | --- | --- | --- | --- |
| **Variable** | **HR (95% CI)** | **P** | **HR (95% CI)** | **P** |
| **Pre-treatment NLR**  < 5  ≥ 5 | 1.0  1.48 (0.91 – 2.42) | --  0.11 | 1.0  1.54 (0.92 – 2.59) | --  0.10 |
| **Age** | 0.99 (0.96 – 1.01) | 0.28 | 1.00 (0.97 – 1.02) | 0.75 |
| **Sex**  Male  Female | 1.0  0.86 (0.55 – 1.36) | --  0.52 | 1.0  0.94 (0.57 – 1.56) | --  0.81 |
| **Clinical Stage**^†^  IIIA  IIIB | 1.0  1.96 (1.24 – 3.09) | --  0.004 | 1.0  1.87 (1.16 – 3.01) | --  0.010 |
| **Histology**  SCC  Adenocarcinoma  NSCLC, NOS | 1.0  1.80 (1.11 – 2.92)  2.65 (1.14 – 6.12) | --  0.017  0.023 | 1.0  2.04 (1.22 – 3.42)  2.64 (1.11 – 6.29) | --  0.006  0.029 |
| **ECOG**  0-1  2-3 | 1.0  1.41 (0.61 – 3.26) | --  0.43 | --  2.13 (0.85 – 5.31) | --  0.11 |
| **Chemotherapy**  Cisplatin/etoposide  Carboplatin/paclitaxel | 1.0  0.89 (0.57 – 1.40) | --  0.61 | 1.0  0.96 (0.57 – 1.61) | --  0.87 |

*Abbreviations: HR- hazard ratio. CI- confidence interval. NLR- neutrophil-lymphocyte ratio. SCC- squamous cell carcinoma. NSCLC, NOS- non-small cell lung cancer, not otherwise specified. ECOG- Eastern Cooperative Oncology Group.

† American Joint Committee on Cancer (AJCC) 7^th^ edition.

**Supplementary Table 3.** Univariate and multivariable hazard ratios for post-treatment NLR at 1 month (post-NLR_1_), analyzed as a continuous variable, for recurrence and survival.

| **Endpoint** | **Univariate** | | **Multivariable** | |
| --- | --- | --- | --- | --- |
|  | **Hazard ratio (confidence interval)** | **P** | **Hazard ratio (confidence interval)** | **P** |
| Overall Survival | 1.04 (1.02 – 1.06) | < .001 | 1.04 (1.02 – 1.06) | <.001 |
| Any Recurrence | 1.03 (1.00 – 1.05) | 0.047 | 1.02 (1.00 – 1.05) | 0.047 |
| Locoregional Recurrence | 1.02 (0.99 – 1.06) | 0.21 | 1.01 (0.97 – 1.06) | 0.54 |
| Distant Recurrence | 1.02 (0.99 – 1.05) | 0.14 | 1.02 (1.00 – 1.05) | 0.089 |

**Supplementary Table 4.** Univariate and multivariable hazard ratios for post-treatment NLR at 1 month (post-NLR_1_) ≥ 6.3 (analyzed as a dichotomized variable), for recurrence and survival.

| **Endpoint** | **Univariate** | | **Multivariable** | |
| --- | --- | --- | --- | --- |
|  | **Hazard ratio (confidence interval)** | **P** | **Hazard ratio (confidence interval)** | **P** |
| Overall Survival | 1.31 (0.86 – 2.00) | 0.21 | 1.25 (0.80 – 1.93) | 0.33 |
| Any Recurrence | 1.56 (1.02 – 2.40) | 0.042 | 1.50 (0.94 – 2.39) | 0.087 |
| Locoregional Recurrence | 1.26 (0.75 – 2.12) | 0.38 | 1.24 (0.71 – 2.16) | 0.44 |
| Distant Recurrence | 1.32 (0.82 – 2.13) | 0.25 | 1.37 (0.82 – 2.28) | 0.23 |

**Supplementary Table 5.** Univariate and multivariable hazard ratios for post-treatment NLR at 3 months (post-NLR_3_), analyzed as a continuous variable, for recurrence and survival.

| **Endpoint** | **Univariate** | | **Multivariable** | |
| --- | --- | --- | --- | --- |
|  | **Hazard ratio (confidence interval)** | **P** | **Hazard ratio (confidence interval)** | **P** |
| Overall Survival | 1.10 (1.07 – 1.14) | < .001 | 1.06 (1.04 – 1.09) | <.001 |
| Any Recurrence | 1.10 (1.06 – 1.14) | < .001 | 1.10 (1.06 – 1.14) | < .001 |
| Locoregional Recurrence | 1.05 (1.01 – 1.09) | 0.011 | 1.07 (1.04 – 1.11) | <.001 |
| Distant Recurrence | 1.04 (1.01 – 1.07) | 0.021 | 1.09 (1.05 – 1.13) | <.001 |

**Supplementary Table 6.** Univariate and multivariable hazard ratios for post-treatment NLR at 3 months (post-NLR_3_) ≥ 6.3 (analyzed as a dichotomized variable), for recurrence and survival.

| **Endpoint** | **Univariate** | | **Multivariable** | |
| --- | --- | --- | --- | --- |
|  | **Hazard ratio (confidence interval)** | **P** | **Hazard ratio (confidence interval)** | **P** |
| Overall Survival | 3.04 (1.91 – 4.84) | <.001 | 3.27 (2.01 – 5.31) | <.001 |
| Any Recurrence | 2.16 (1.37 – 3.38) | <.001 | 2.50 (1.53 – 4.08) | <.001 |
| Locoregional Recurrence | 2.21 (1.29 – 3.81) | 0.004 | 2.50 (1.40 – 4.46) | 0.002 |
| Distant Recurrence | 1.93 (1.19– 3.13) | 0.008 | 2.53 (1.49 – 4.30) | <.001 |

**Supplementary Figure 1.** Diagram of analyzed patients included in the study.

**Supplementary Figure 2.** Boxplot of NLR values before treatment and at 1 month and 3 months after treatment. Pre-treatment vs 1 month post treatment Wilcoxon signed rank p < 0.001. Pre-treatment vs 3 months post-treatment Wilcoxon signed rank p < 0.001. One month post-treatment vs 3 months post-treatment Wilcoxon signed rank p = 0.014. Bonferonni corrected α = 0.05 / 3 = 0.017.


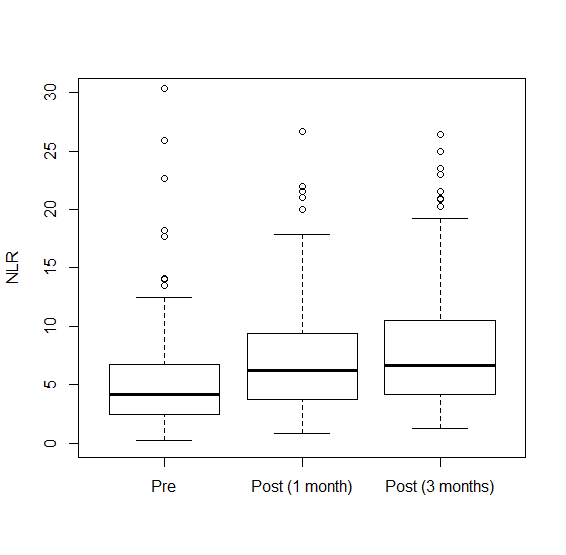


**Supplementary Figure 3.** Kaplan-Meier curves for **(A)** overall survival **(B)** freedom from recurrence **(C)** freedom from locoregional recurrence, and **(D)** freedom from distant recurrence, stratified by 1-month-post-treatment NLR (Post-NLR_1_) < 6.3 and ≥ 6.3.

**(A)**


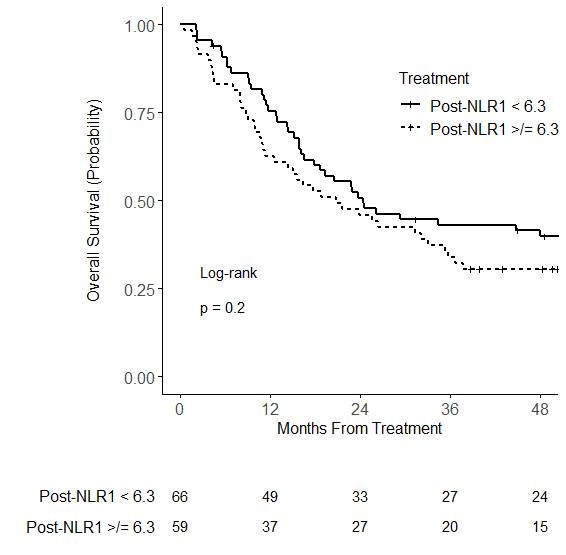


**(B)**


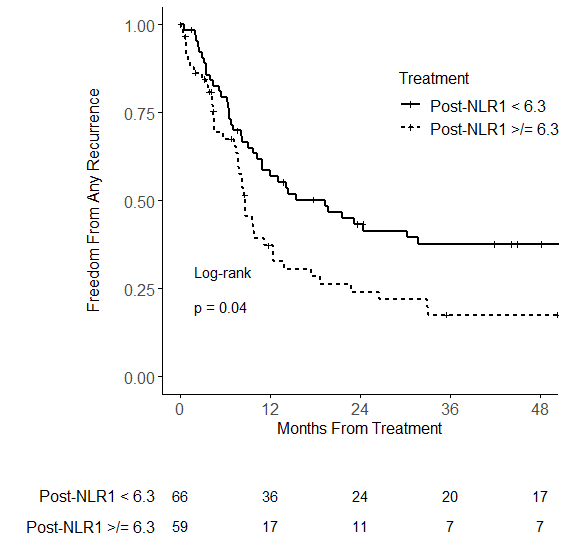


**(C)**


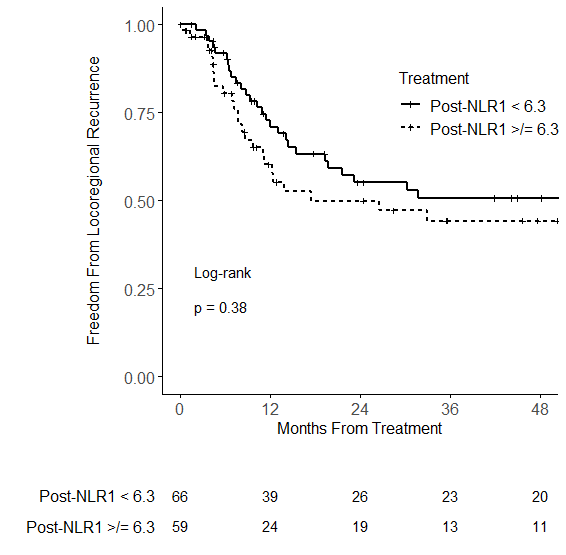


**(D)**


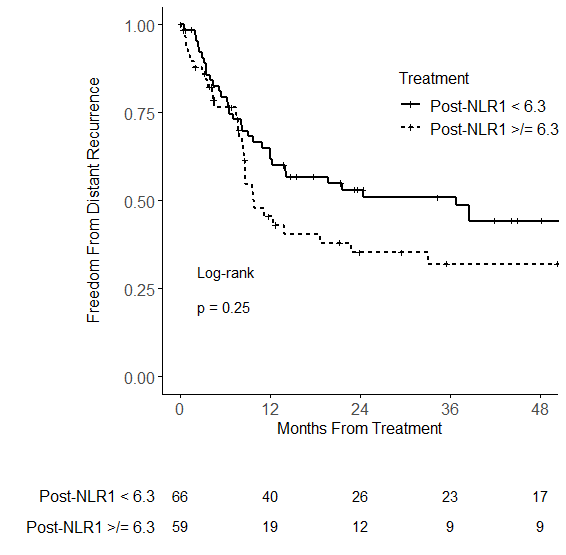

Supplement: Supplementary file 1 [file Table_1.docx]
